# Symmetry-protected bound states in the continuum on an integrated photonic platform

**DOI:** 10.1515/nanoph-2024-0196

**Published:** 2024-08-02

**Authors:** Qijing Lu, Ziyao Feng, Xiankai Sun

**Affiliations:** Department of Electronic Engineering, The Chinese University of Hong Kong, Shatin, New Territories, Hong Kong SAR, China; Key Laboratory of Optoelectronic Science and Technology for Medicine of Ministry of Education, Provincial Key Laboratory for Photonics Technology, Fujian Normal University, Fuzhou 350007, China

**Keywords:** bound states in the continuum, integrated photonics, symmetry-protected, optical microcavities, filters

## Abstract

Bound states in the continuum (BICs) have attracted much attention in the field of nanophotonics owing to their ability to trap photons without loss. Recently, a low-refractive-index (RI) waveguide loaded on a high-RI slab structure was demonstrated to support BICs. However, strict control of structural parameters is required due to the accidental nature of those BICs. Here, we propose a novel structure consisting of two low-RI vertically coupled waveguides loaded on a high-RI slab. This structure supports symmetry-protected BICs (SP-BICs), which do not require strict control of the geometric parameters. Such SP-BICs can also possess an infinitely high quality factor in resonant structures, which can be harnessed for ultranarrow-bandwidth spatial and spectral filters. Our work opens a new way of harnessing BICs on an integrated photonic platform for realizing nanophotonic circuits and devices.

## 1 Introduction

In optical systems, bound states traditionally refer to modes where photons are confined by mechanisms such as reflection, refraction, or absorption within microcavities, fibers, or other resonant optical structures. These modes have discrete frequencies and can interact with the radiation modes when their frequencies fall within the continuous spectrum, resulting in degradation of the photon lifetime (or equivalently quality factor, *Q*) and overall device performance. By contrast, bound states in the continuum (BICs) [1], [2] are localized and nonradiating states with infinite photon lifetime despite being surrounded by a series of unbound continuous states. The concept of BIC was first proposed as a mathematical solution in quantum mechanics [3], and its fascinating features have been widely investigated in photonics [4], [5], [6], atomic physics [7], acoustics [5], [8], [9], and hydrodynamics [10] in recent years.

BICs are typically classified into symmetry-protected BICs (SP-BICs) and accidental BICs [2], [11]. The former is formed by a mismatch between the spatially symmetric mode of the BIC and the outgoing radiating mode, which effectively blocks all the leakage channels for the BIC [12]. The latter is formed through destructive interference among different coupled modes, which is achieved by tuning system parameters to cancel out the radiating components. It should be noted that the boundary between the two classes of BICs is not always clear, as SP-BIC can be interpreted as the presence of destructive interference in certain scenarios [2], [13]. BICs have been realized successfully on various photonic platforms, including photonic crystal slabs [11], [12], [14], [15], metasurfaces [16], and waveguides [4]. One type of BIC structures that has garnered significant attention consists of a single waveguide with a low refractive index (RI) on a thin membrane with a high RI [17], [18], [19], [20]. This structure offers advantages such as scalability for the development of photonic integrated circuits and compatibility with etchless fabrication processing, and thus has been employed in a wide range of applications including nonlinear optics [21], [22], [23], optical communications [24], spectrometers [19], [20], and quantum networks [17]. In a single-waveguide structure, the BIC is achieved by destructive interference among various channels of power leakage from the transverse magnetic (TM) bound mode in the waveguide to the transverse electric (TE) continuous mode in the slab [17]. This BIC is considered accidental and occurs only at specific waveguide widths, referred to as the “magic widths” [25]. Recently, there are studies of BICs in structures with two horizontally coupled waveguides on the slab [26], [27], [28], which can support BICs with arbitrary waveguide widths. However, in this case, the distance between the two waveguides becomes another critical parameter [28]. In this regard, the realization of accidental BICs in these horizontally coupled waveguides still relies on some strictly designed structural parameters.

Here, we propose a general approach to realize robust BICs on such low-RI-waveguide-on-high-RI-slab integrated photonic platforms. By arranging two waveguides symmetrically on both sides of the slab, this structure supports SP-BICs without the condition of strictly designed structural parameters, such as waveguide width and separation. Furthermore, such SP-BICs can be adopted in two types of resonant structures: microring and ridge resonators. These structures exhibit ultrahigh-*Q* resonances, which can be used in applications of ultrasensitive sensing and ultranarrow-bandwidth filtering. The combination of the SP-BICs and the resonant structures opens a new way for high-performance photonic devices.

## 2 Results and discussion

**Figure 1:**
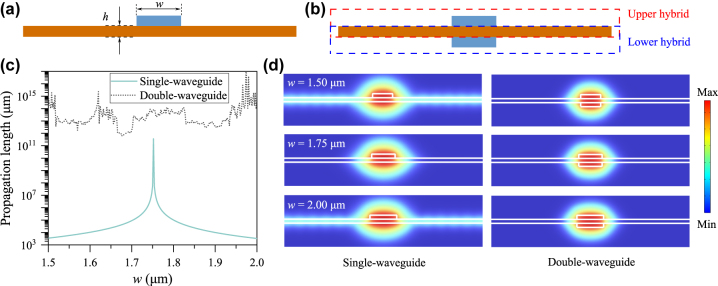
(a, b) Schematics of the conventional single-waveguide (a) and the proposed double-waveguide (b) loaded on a high-RI slab. (c) Propagation length of the supported bound mode as a function of the waveguide width *w*. (d) Modal profiles (|**E**| field component plotted on a logarithmic scale) supported by the single-waveguide (left) and double-waveguide (right) structures with *w* = 1.50 μm, 1.75 μm, and 2.00 μm.

Figure 1(a) illustrates a conventional single low-RI waveguide on a high-RI slab, where *w* and *h* denote the waveguide width and thickness, respectively. Without loss of generality, we choose the operating wavelength as 1,550 nm, the RI of the waveguide as 1.5 (a value close to that of polymer or silica), and the RI of the slab as 2.0 (a value close to that of lithium niobate or silicon nitride). The thickness of the slab *h* is set as 300 nm throughout this study. In this single-waveguide configuration, the guided modes of the waveguide are coupled with the radiation modes of the slab, forming hybrid bound modes. The TM-polarized hybrid bound modes are more favored than the TE-polarized counterparts because the former can have tighter light confinement and smaller modal areas. Moreover, because the RI of the waveguide is lower than that of the slab, the TM-polarized hybrid bound modes lie within the continuous spectrum of the TE-polarized hybrid radiation modes. At specific combinations of the structural parameters, the TM-polarized hybrid bound mode can become lossless and form an accidental BIC [17], [18].

Figure 1(b) illustrates the proposed double-waveguide structure where a high-RI slab is sandwiched by two identical low-RI waveguides arranged vertically, which can support SP-BICs. In this double-waveguide configuration, since the guided modes of both waveguides can be coupled with the radiation modes of the slab, the system can be modeled as coupling between the upper hybrid bound mode and the lower hybrid bound mode, with an effective Hamiltonian

(1) $$\hat{H}=\left(\begin{array}{cc} \beta_{u}-i\gamma_{u} & \tilde{\kappa }\\ \tilde{\kappa } & \beta_{l}-i\gamma_{l} \end{array}\right),$$

where *β*_*u*(*l*)_ and *γ*_*u*(*l*)_ are the eigenfrequency and dissipation rate, respectively, of the uncoupled upper (lower) hybrid bound mode, and $\tilde{\kappa }=\kappa -i\sqrt{\gamma_{u}\gamma_{l}}$ is the coupling strength. The eigenvalues of the Hamiltonian in Eq. (1) are

(2) $$\begin{array}{ll} {\tilde{\beta }}_{\pm } & =\frac{\beta_{u}-i\gamma_{u}+\beta_{l}-i\gamma_{l}}{2}\\ & \;\pm \sqrt{{\left[\left(\beta_{u}-i\gamma_{u}\right)-\left(\beta_{l}-i\gamma_{l}\right)\right]}^{2}/4+{\tilde{\kappa }}^{2}}. \end{array}$$

As the two waveguides are identical, one has *β*_*u*_ = *β*_*l*_ = *β*_*h*_, *γ*_*u*_ = *γ*_*l*_ = *γ*_*h*_, so Eq. (2) can be simplified as

(3) $$\left\{\begin{array}{c} {\tilde{\beta }}_{+}=\beta_{h}+\kappa -2\gamma_{h}i,\;\\ {\tilde{\beta }}_{-}=\beta_{h}-\kappa \cdot \; \end{array}\right.$$

It is obvious that one of the resulting two modes is lossy, and the other is lossless. Therefore, a BIC is always guaranteed in this symmetric structure.

We adopted a finite-element method (COMSOL Multiphysics) to simulate the waveguide structures and analyze their modal properties. The propagation length of a mode, which is inversely proportional to its loss rate, was calculated as $L=\lambda /\left[4\pi \text{Im}\left(n_{\text{eff}}\right)\right]$ , with *n*_eff_ being the complex effective RI. Figure 1(c) plots the simulated propagation length of the bound mode as a function of the waveguide width *w* for both conventional single-waveguide (solid line) and proposed double-waveguide (dotted line) structures. Figure 1(d) shows the modal profiles (|**E**| field component plotted on a logarithmic scale) for the single-waveguide (left) and double-waveguide (right) structures. For the single-waveguide structure, the propagation length of the supported bound mode as shown in Figure 1(c) reaches a maximum at a specific *w* value, which confirms that the BIC is achieved under a stringent (though experimentally achievable) condition, such as *w* = 1.75 μm in this numerical example. The modal profiles in the left column of Figure 1(d) provide a more intuitive picture of this phenomenon. Under off-BIC conditions, such as *w* = 1.50 μm or *w* = 2.00 μm, the bound mode is coupled to the slab’s continuous modes, and thus is lossy due to the lateral power leakage. By contrast, under the BIC condition of *w* = 1.75 μm, the bound mode becomes lossless and possesses an infinite lifetime due to destructive interference among all the power leakage channels. For the double-waveguide structure, the propagation length of the supported bound mode as shown in Figure 1(c) maintains extremely large for all waveguide widths. Note that the undulating behavior of the propagation length for the double-waveguide structure in Figure 1(c) is caused by precision limitation of the finite-element method in the simulation, which does not have any physical significance. The modal profiles in the right column of Figure 1(d) indicate that the bound mode is always lossless as it is free from any lateral power leakage. This also confirms that the SP-BIC is supported in the double-waveguide structure and does not rely on some specific waveguide widths.

**Figure 2:**
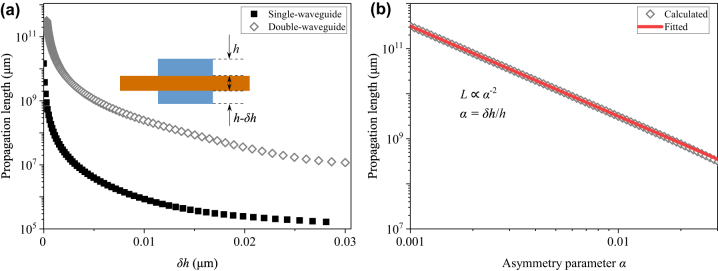
(a) Propagation length as a function of the perturbation *δh*. The inset shows the description of the perturbation. (b) Propagation length as a function of the asymmetry parameter *α*.

The ideal double-waveguide structure supports a SP-BIC with an infinite propagation length. When the plane symmetry is broken, the ideal BIC becomes a quasi-BIC with a finite propagation length. Next, we studied the impact of a perturbation *δh* to the thickness of one of the double waveguides (see Figure 2(a) inset) on the quasi-BIC. Figure 2(a) plots the simulated propagation length as a function of *δh* with *w* = 1.75 μm and *h* = 300 nm. For comparison, Figure 2(a) also includes the quasi-BIC’s propagation length as a function of *δh* for the single-waveguide structure. It is clear that, when *δh* = 0, the propagation length is infinite for both structures. A linear increase of *δh* results in an exponential decay of the propagation length for both structures. However, with the same *δh*, the quasi-BIC’s propagation length in the double-waveguide structure is consistently orders of magnitude larger than that in the single-waveguide structure. This confirms the robustness of the proposed double-waveguide structure, even though it possesses structural perturbations breaking the plane symmetry. Figure 2(b) plots the quasi-BIC’s propagation length in the asymmetric double-waveguide structure as a function of the asymmetry parameter *α* (*α* = *δh*/*h*). It is clear that the propagation length is proportional to *α* ^−2^, which follows the universal scaling law and satisfies the criterion for SP-BICs [29].

**Figure 3:**
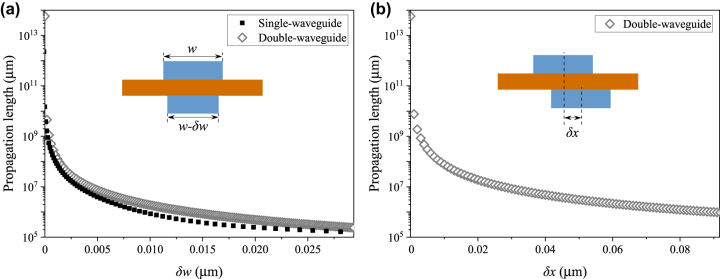
(a) Propagation length as a function of the width difference *δw* between the double waveguides. (b) Propagation length as a function of the center misalignment *δx* between the double waveguides.

We studied two other types of perturbations to the double-waveguide structure. The first type is a width difference *δw* between the double waveguides with both waveguides horizontally centered and aligned, as shown in Figure 3(a) inset. Figure 3(a) shows that the double-waveguide structure outperforms the single-waveguide structure at the same *δw*. The second type is a center misalignment *δx* between the double waveguides with the widths of the two waveguides being equal, as shown in Figure 3(b) inset. Both Figure 3(a) and (b) show an exponential decay of the propagation length with a linear increase of *δw* or *δx*. This decay is due to the breaking of structural symmetry. For the propagation length to be larger than 1 m, the perturbations *δw* and *δx* have to be controlled within approximately ±15 nm and ±90 nm, respectively.

**Figure 4:**
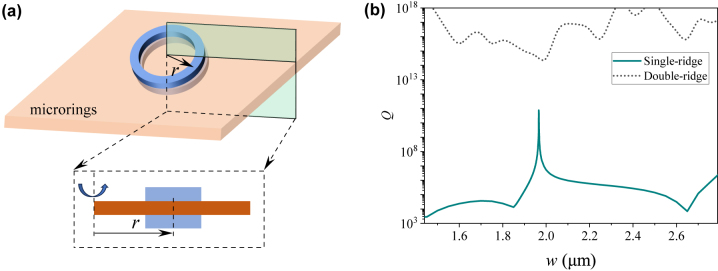
(a) Schematic of the symmetric double-microring. (b) *Q* factors as a function of the ring width *w*.

To enhance light–matter interaction, we investigated two types of resonant structures constructed from the symmetric double waveguides. The first type of resonant structure is a whispering-gallery-mode (WGM) microring cavity, as depicted in Figure 4(a). In such WGM microring cavities, the loss consists of two contributions: power leakage to the continuous modes of the slab and bending loss to the radiation modes of the free space. Here, we chose a relatively large ring radius *r* = 50 μm such that the bending loss can be neglected. We simulated the WGM microring cavities constructed from single- and double-waveguide structures with a two-dimensional axisymmetric model and obtained their resonant eigenfrequencies *f* and the corresponding cavity *Q* factors [ $Q=\text{Re}\left(f\right)/2\text{Im}\left(f\right)$ ] as a function of the ring width *w*, as plotted in Figure 4(b). For the single-microring cavity, the condition for the BIC is achieved at a specific ring width of *w* = 1.98 μm. By contrast, for the double-microring cavity, the SP-BIC is always obtained regardless of the ring width. Here, the simulated *Q* factor is determined by the power leakage to the continuous modes and the bending loss to the radiation modes. When the material absorption loss is considered, the *Q* factor is not infinite any more. If the low-RI waveguide is made of silica and the high-RI slab is made of silicon nitride, then the absorption loss is dominated by silicon nitride, as the absorption coefficient of silicon nitride is two orders of magnitude higher than that of silica. The absorption coefficient *α* of silicon nitride is ∼0.0001 cm^−1^, resulting in an absorption-limited quality factor *Q*_abs_ $\left[=2\pi n_{\text{eff}}/\left(\alpha \lambda \right)\right]$ of ∼5 × 10^8^. Therefore, under the BIC condition, the *Q* factors for both single-ring and double-ring structures are eventually determined by the absorption loss when the scattering loss can be ignored.

**Figure 5:**
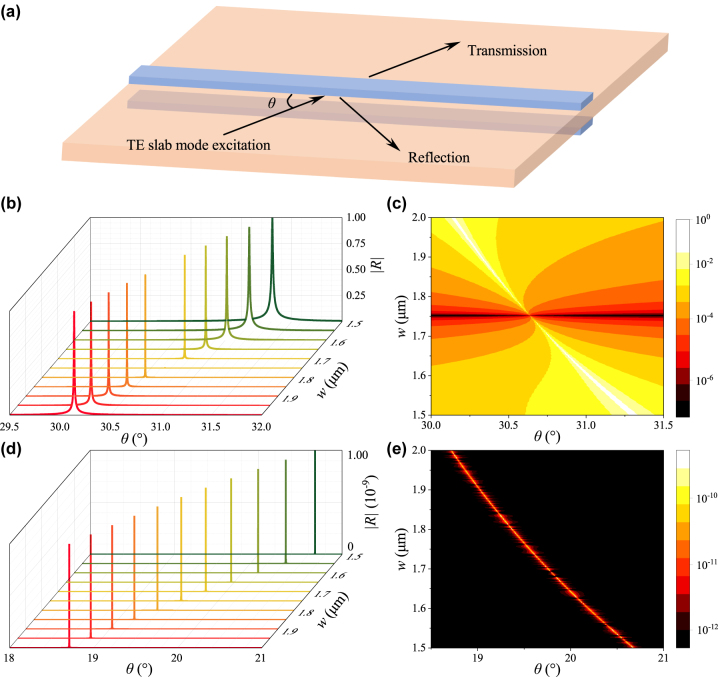
(a) Schematic of the excitation scheme for the double-ridge structure. (b, d) Angular reflection spectra of the single-ridge structure (b) and double-ridge structure (d) as a function of the ridge width *w* and incidence angle *θ*. (c, e) Corresponding contour maps of the angular reflection spectra for the single-ridge structure (c) and double-ridge structure (e).

The second type of resonant structure is based on ridge waveguides under excitation of a cross-polarized mode [25], [30], [31], [32]. Here, we construct the double-ridge structure from the symmetric double waveguides and investigate its spatial (angular) and spectral resonance properties. Figure 5(a) shows the excitation scheme for the double-ridge structure. A TE slab mode is launched at an oblique angle *θ* to excite a TM guided mode in the ridge. With carefully designed structural geometry, it is possible to support a single mode in both TM and TE polarizations [31]. Here, we focus on the fundamental TM and TE modes. Since the TM bound mode in the ridge can radiate into a TE slab mode at an oblique angle on both sides, conversely, it can resonantly be excited by the TE slab mode launched at the same oblique angle. Neglecting nonresonant reflections, the reflection coefficient can be expressed as [25]

(4) $$R=\frac{-i\text{Im}\left(n_{\text{eff},\text{TM}}\right)}{i\left[n_{\text{eff},\text{TE}}\cos\theta -\text{Re}\left(n_{\text{eff},\text{TM}}\right)\right]+\text{Im}\left(n_{\text{eff},\text{TM}}\right)}.$$

It is clear from Eq. (4) that when $n_{\text{eff},\text{TE}}\cos\theta =\text{Re}\left(n_{\text{eff},\text{TM}}\right)$ , the resonant condition is satisfied and the TE slab mode is totally reflected. The angular width of the resonance is determined by the loss of the TM bound mode, which is associated with the ridge width. When the BIC is achieved, the TM bound mode becomes lossless, resulting in an infinitely narrow angular linewidth for the resonance. At the resonance, the TM bound mode is decoupled from all the TE continuous modes of the slab, and thus the launched TE slab mode can propagate across the ridge perfectly without any loss.

Figure 5(b) shows the angular reflection spectra for the single-ridge structure with several ridge widths. The resonance occurs at a specific *θ* depending on the ridge width *w* due to structural dispersion of the TM bound mode. When *w* = 1.75 μm, the resonance achieves an infinite *Q* factor, and correspondingly the reflection peak disappears. The disappearance of a prominent reflection peak results from the presence of BIC for the TM bound mode. As *w* deviates from 1.75 μm, the *Q* factor decreases exponentially. Figure 5(c) shows the contour map of the reflection spectra (logarithmic scale) as a function of the incident angle *θ* and ridge width *w*. It is clear that the BIC is achieved at *θ* = 30.6° and *w* = 1.75 μm. Figure 5(d) and (e) show the angular reflection spectra and the contour map, respectively, for the double-ridge structure. Note that due to the extremely high *Q* of the resonance, its peak reflectance remains small for all the *w* values. To identify the resonant angle corresponding to the BIC, Figure 5(d) shows a close-up view in the *R* range of [0, 10^−9^]. For all the *w* values, the resonance achieves an infinite *Q* factor, confirming the presence of the SP-BIC. In contrast to the “BIC point” shown in the contour map of reflection spectra of the single-ridge structure [Figure 5(c)], the double-ridge structure supports a “BIC line” in its contour map of reflection spectra [Figure 5(e)].

**Figure 6:**
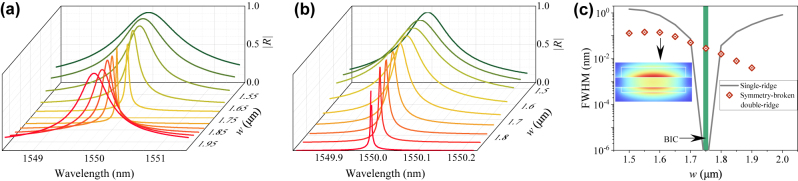
(a, b) Reflection spectra of the single-ridge structure (a) and symmetry-broken double-ridge structure (b) with different ridge widths *w*. (c) FWHM of the reflection peak as a function of the ridge width *w*. Inset: Modal profile (|**E**| component) of the quasi-BIC in the symmetry-broken double-ridge structure.

The angular reflection spectra of the double-ridge structure exhibit a peak with a Lorentzian line shape, which makes this structure suitable for development of ultranarrow-bandwidth spatial and angular filters. Due to the structural dispersion of the TM bound mode and TE slab mode, the reflectance and the BIC condition are also wavelength-dependent. Figure 6 presents the reflection spectra of the single-ridge and double-ridge structures for several *w* values and the full width at half maximum (FWHM) of the reflection peaks. Considering the experimental realisticity, we used a symmetry-broken double-ridge structure in the simulation, where the thickness of the lower ridge is set as 150 nm (different from 300 nm for the upper ridge). This symmetry-broken structure supports a quasi-BIC, with its modal profile (|**E**| component) shown in the inset of Figure 6(c). For the single-ridge structure, the FWHM of the reflection peak is sensitive to the *w* value and varies drastically near the BIC condition, as shown in Figure 6(a). Actually, when the BIC condition is satisfied at *w* = 1.75 μm, the reflection peak disappears with a zero FWHM. By contrast, for the symmetry-broken double-ridge structure, the FWHM of the reflection peak decreases monotonically and more gradually as *w* increases, as shown in Figure 6(b) and (c). Therefore, it is possible to obtain a filter with a more controllable and predictable bandwidth by using a symmetry-broken double-ridge structure with a designed ridge width.

Finally, we suggest three potential methods for fabricating the double-waveguide structures on a lithium-niobate-on-insulator platform. The first method involves a standard lithography process. First, the silica beneath the lithium niobate (LN) thin film is selectively etched by using hydrofluoric acid [15]. Then, a polymer (ZEP520A) is spin-coated on the substrate to form both lower and upper claddings for the LN thin film. Last, a single step of electron-beam lithography is performed to pattern the double polymer waveguides [18], which produces identical widths for the top and bottom waveguides and guarantees the plane symmetry of the structure. The second method involves femtosecond laser writing of waveguides [33]. First, a thick layer of silica is deposited on the lithium-niobate-on-insulator substrate. Then, a femtosecond laser is focused to the central plane of the LN thin film to locally modify the refractive indices of both the LN and silica, which forms the symmetric double silica waveguides. The third method is based on the recently reported heterogeneously integrated LN photonic circuits [34], [35]. A silicon nitride film is deposited on heterogeneously integrated LN photonic circuits by chemical vapor deposition and is then patterned by reactive ion etching to create the waveguide or microring. Next, silica is deposited as the top cladding by plasma-enhanced chemical vapor deposition. The alignment between the upper and lower waveguides can be guaranteed in the lithographic process with carefully designed alignment marks and calibrators [36].

## 3 Conclusions

In conclusion, we have theoretically proposed and numerically verified a type of SP-BICs on an integrated photonic platform. These SP-BICs are supported by two vertically coupled low-RI waveguides loaded on a high-RI slab. The satisfaction of the BIC condition depends only on the plane symmetry of the structure and not on the waveguide geometric parameters. The numerical simulations confirmed the existence of SP-BICs and demonstrated their robust properties even in the symmetry-broken structures. These SP-BICs can also be employed in resonant structures such as microring cavities and ridge structures, to obtain infinite *Q* factors with enhanced light–matter interaction. The proposed structure can also be adopted for realizing an etchless photonic crystal LN thin-film heteronanostructure [23], where an etchless LN film sandwiched between two low-RI photonic crystal structures can support robust SP-BICs with localized field confinement. We also suggested potential methods for fabricating the symmetric double-waveguide structures. These SP-BICs may be harnessed for exploring non-Hermitian physics, such as exceptional points, nonreciprocity, and topological effects [26], [27]. This work may also be harnessed for developing new generations of photonic circuits and devices, such as ultrasensitive sensors and ultranarrow-bandwidth filters [2].

## References

[1] Hsu C. W., Zhen B., Stone A. D., Joannopoulos J. D., Soljačić M. (2016). Bound states in the continuum. *Nat. Rev. Mater.*.

[2] Kang M., Liu T., Chan C., Xiao M. (2023). Applications of bound states in the continuum in photonics. *Nat. Rev. Phys.*.

[3] Neumann J. V., Wigner E. (1929). Über merkwürdige diskrete eigenwerte. *Phys. Z*.

[4] Plotnik Y. (2011). Experimental observation of optical bound states in the continuum. *Phys. Rev. Lett.*.

[5] Huang L. (2021). Sound trapping in an open resonator. *Nat. Commun.*.

[6] Wang J. (2024). Optical bound states in the continuum in periodic structures: mechanisms, effects, and applications. *Photon. Insights*.

[7] Guessi L. (2015). Catching the bound states in the continuum of a phantom atom in graphene. *Phys. Rev. B*.

[8] Lyapina A., Maksimov D., Pilipchuk A., Sadreev A. (2015). Bound states in the continuum in open acoustic resonators. *J. Fluid Mech.*.

[9] Jia B. (2023). Bound states in the continuum protected by reduced symmetry of three-dimensional open acoustic resonators. *Phys. Rev. Appl.*.

[10] Cobelli P., Pagneux V., Maurel A., Petitjeans P. (2011). Experimental study on water-wave trapped modes. *J. Fluid Mech.*.

[11] Hsu C. W. (2013). Observation of trapped light within the radiation continuum. *Nature*.

[12] Zhen B., Hsu C. W., Lu L., Stone A. D., Soljačić M. (2014). Topological nature of optical bound states in the continuum. *Phys. Rev. Lett.*.

[13] Hu P. (2022). Global phase diagram of bound states in the continuum. *Optica*.

[14] Huang C. (2020). Ultrafast control of vortex microlasers. *Science*.

[15] Kodigala A., Lepetit T., Gu Q., Bahari B., Fainman Y., Kanté B. (2017). Lasing action from photonic bound states in continuum. *Nature*.

[16] Koshelev K., Tonkaev P., Kivshar Y. (2023). Nonlinear chiral metaphotonics: a perspective. *Adv. Photonics*.

[17] Zou C. L. (2015). Guiding light through optical bound states in the continuum for ultrahigh-*Q* microresonators. *Laser Photonics Rev.*.

[18] Yu Z., Xi X., Ma J., Tsang H. K., Zou C.-L., Sun X. (2019). Photonic integrated circuits with bound states in the continuum. *Optica*.

[19] Pohl D. (2020). An integrated broadband spectrometer on thin-film lithium niobate. *Nat. Photonics*.

[20] Chen L. (2023). Demonstration of a high-performance 3 dB power splitter in silicon nitride loaded lithium niobate on insulator. *Laser Photonics Rev.*.

[21] Ye F., Yu Y., Xi X., Sun X. (2022). Second‐harmonic generation in etchless lithium niobate nanophotonic waveguides with bound states in the continuum. *Laser Photonics Rev.*.

[22] Li X. (2022). Efficient second harmonic generation by harnessing bound states in the continuum in semi-nonlinear etchless lithium niobate waveguides. *Light Sci. Appl.*.

[23] Huang Z. (2022). Resonant enhancement of second harmonic generation in etchless thin film lithium niobate heteronanostructure. *Sci. China Phys. Mech. Astron.*.

[24] Yu Z., Tong Y., Tsang H. K., Sun X. (2020). High-dimensional communication on etchless lithium niobate platform with photonic bound states in the continuum. *Nat. Commun.*.

[25] Nguyen T. G., Ren G., Schoenhardt S., Knoerzer M., Boes A., Mitchell A. (2019). Ridge resonance in silicon photonics harnessing bound states in the continuum. *Laser Photonics Rev.*.

[26] Feng Z., Sun X. (2023). Experimental observation of dissipatively coupled bound states in the continuum on an integrated photonic platform. *Laser Photonics Rev.*.

[27] Qin H., Shi X., Ou H. (2022). Exceptional points at bound states in the continuum in photonic integrated circuits. *Nanophotonics*.

[28] Gu Z., Jiang S., Liu C., Zhang N. (2023). Robust bound states in the continuum in a dual waveguide system. *Photonics Res.*.

[29] Koshelev K., Lepeshov S., Liu M., Bogdanov A., Kivshar Y. (2018). Asymmetric metasurfaces with high-*Q* resonances governed by bound states in the continuum. *Phys. Rev. Lett.*.

[30] Hammer M., Hildebrandt A., Förstner J. (2015). How planar optical waves can be made to climb dielectric steps. *Opt. Lett.*.

[31] Bezus E. A., Bykov D. A., Doskolovich L. L. (2018). Bound states in the continuum and high-*Q* resonances supported by a dielectric ridge on a slab waveguide. *Photonics Res.*.

[32] Doskolovich L. L., Bezus E. A., Bykov D. A. (2019). Integrated flat-top reflection filters operating near bound states in the continuum. *Photonics Res.*.

[33] Zhang Z., Li Y., Sun X., Shu X. (2024). Visual observation of photonic Floquet–Bloch oscillations. *Light Sci. Appl.*.

[34] Churaev M. (2023). A heterogeneously integrated lithium niobate-on-silicon nitride photonic platform. *Nat. Commun.*.

[35] Han K., LeBrun T. W., Aksyuk V. A. (2024). Bound-state-in-continuum guided modes in a multilayer electro-optically active photonic integrated circuit platform. *Optica*.

[36] Yoon G., Kim I., So S., Mun J., Kim M., Rho J. (2017). Fabrication of three-dimensional suspended, interlayered and hierarchical nanostructures by accuracy-improved electron beam lithography overlay. *Sci. Rep.*.

